# Studies on Haloperidol and Adjunctive α-Mangostin or Raw *Garcinia mangostana* Linn Pericarp on Bio-Behavioral Markers in an Immune-Inflammatory Model of Schizophrenia in Male Rats

**DOI:** 10.3389/fpsyt.2020.00121

**Published:** 2020-03-31

**Authors:** Jana Lotter, Marisa Möller, Olivia Dean, Michael Berk, Brian H. Harvey

**Affiliations:** ^1^Division of Pharmacology, Center of Excellence for Pharmaceutical Sciences, School of Pharmacy, North West University, Potchefstroom, South Africa; ^2^Deakin University, IMPACT - The Institute for Mental and Physical Health and Clinical Translation, School of Medicine, Barwon Health, Geelong, Australia; ^3^Florey Institute of Neuroscience and Mental Health, The University of Melbourne, Parkville, VIC, Australia; ^4^Orygen, Department of Psychiatry, The Centre of Excellence in Youth Mental Health, The University of Melbourne, Parkville, VIC, Australia

**Keywords:** antidepressant, oxidative stress, immunity, antipsychotic, complimentary medicine, maternal inflammation, mangosteen, adjunctive treatment

## Abstract

Schizophrenia is a severe brain disorder that is associated with neurodevelopmental insults, such as prenatal inflammation, that introduce redox-immune-inflammatory alterations and risk for psychotic symptoms later in life. Nutraceuticals may offer useful adjunctive benefits. The aim of this study was to examine the therapeutic effects of *Garcinia mangostana* Linn (GML) and one of its active constituents, α-mangostin (AM), alone and as adjunctive treatment with haloperidol (HAL) on schizophrenia related bio-behavioral alterations in a maternal immune-activation (MIA) model. Sprague–Dawley dams were exposed to lipopolysaccharide (LPS) (*n* = 18) or vehicle (*n* = 3) on gestational days 15 and 16. Male offspring (*n* = 72) were treated from PND 52–66 with either vehicle, HAL (2 mg/kg), GML (50 mg/kg), HAL + GML, AM (20 mg/kg), or HAL + AM. Control dams and control offspring were treated with vehicle. In order to cover the mood–psychosis continuum, prepulse inhibition (PPI) of startle, open field test (locomotor activity), and the forced swim test (depressive-like behavior) were assessed on PND's 64–65, followed by assay of frontal–cortical lipid peroxidation and plasma pro-inflammatory cytokines, *viz*. interleukin-1 (IL-1) and tumor necrosis factor-α (TNF-α). MIA-induced deficits in sensorimotor gating were reversed by HAL and HAL + GML, but not GML and AM alone. MIA-induced depressive-like behavior was reversed by AM and GML alone and both in combination with HAL, with the combinations more effective than HAL. MIA-induced cortical lipid peroxidation was reversed by HAL and AM, with elevated IL-6 levels restored by GML, AM, HAL, and HAL + GML. Elevated TNF-α was only reversed by GML and HAL + GML. Concluding, prenatal LPS-induced psychotic- and depressive-like bio-behavioral alterations in offspring are variably responsive to HAL, GML, and AM, with depressive (but not psychosis-like) manifestations responding to GML, AM, and combinations with HAL. AM may be a more effective antioxidant than GML *in vivo*, although this does not imply an improved therapeutic response, for which trials are required.

## Introduction

Schizophrenia is a severe psychiatric disorder with a chronic course, affecting ~1% of the global population (1). This debilitating disease manifests in early adulthood (2), presenting with positive (hallucinations and delusions), negative (social withdrawal, apathy, and anhedonia), and cognitive symptoms (working memory deficits, attention disorders, and altered information processing) (3). However, the underlying etiological mechanisms remain elusive (4). Similarly, the treatment outcome for schizophrenia remains suboptimal (5), especially with regard to negative and cognitive symptoms (6, 7).

The neurodevelopmental hypothesis has provided a valuable framework for establishing the relationship between pathologic processes during early brain development and the development of schizophrenia later in life (8, 9). This hypothesis suggests an interaction between genetic predisposition and early life environmental vulnerability factors such as malnutrition, substance abuse, obstetric complications, season of birth and infection, and exacerbation by later stresses such as substance abuse, social defeat, and trauma (10–13).

Viral or bacterial maternal infection during pregnancy has been linked to increased risk for developing schizophrenia in the offspring (14, 15), with immune activation rather than the infectious agent, itself, being deemed causal (16, 17). Indeed, trauma is associated with immune activation and increased risk of psychosis (18). Immune adjuvants such as lipopolysaccharide (LPS) (19–21), polyinosinic:polycytidylic acid (poly I:C) (22–24), human influenza virus (25), and cytokines (26, 27) induce diverse biological and behavioral abnormalities in rodents following prenatal maternal exposure. LPS, an endotoxin derived from the cell wall of Gram-negative bacteria, mimics an infection by activating the synthesis and release of pro-inflammatory cytokines, including interleukin-1β (IL-1b), IL-6, and tumor necrosis factor-α (TNF-α) (28–30) and engenders various schizophrenia-like behavioral, neurochemical, and inflammatory changes (31–33).

Oxidative stress underscores various psychiatric conditions (34, 35), in particular, schizophrenia (36). Increased reactive oxygen species (ROS) and reduced antioxidants observed in schizophrenia may contribute to the neuroprogression of the disorder (37) and to the development of cognitive dysfunction (38, 39). Indeed, the antioxidant, N-acetyl cysteine (NAC), has therapeutic benefits in various clinical domains of schizophrenia, but especially negative symptoms (40, 41) and cognition (42), while having also demonstrated efficacy in preclinical animal models (43–45).

There is an increased drive to integrate nutraceuticals and psychotropic herbal medicines into conventional medical practice (46). Co-prescription of certain herbal medicines with traditional pharmaceuticals may display complementary pharmacodynamic actions and so provide a beneficial synergistic effect (46, 47). However, little study has occurred that has directly explored such augmentation effects (46). With raw herbal extracts containing a vast array of potentially bioactive ingredients, the question remains whether the observed pharmacological effect is ingredient specific or a sum effect of the total extract.

The anti-inflammatory and antioxidant activities of herbal bioactive compounds have been widely observed, particularly in a group of polyphenols referred to as xanthones (48). *Garcinia mangostana* Linn (GML) is a fruit native to Southeast Asia known to contain constituents including xanthones, flavonoids, triterpenoids, and benzophenones (49). Extracts of the fruit have exhibited antioxidant (50, 51), anti-inflammatory (52, 53), antibacterial (54), and antidepressant effects (55). In particular, α-mangostin (AM), a primary component of GML, presents with substantial pharmacological properties (56, 57), including antioxidant activity (58), as well as having moderate inhibitory effects on 5HT_2A_ receptors and cyclic adenosine monophosphate (cAMP) phosphodiesterase (PDE) (49), actions that hint at possible clinical utility as a pharmacological intervention in psychiatric disorders.

The aim of this study was to establish whether maternal immune activation (MIA) induced schizophrenia-like behavior and redox-inflammatory alterations in offspring can be reversed with the typical antipsychotic, haloperidol (HAL), GML, and AM separately. Second, since the most common use for nutraceuticals in clinical psychiatry is as an adjunctive treatment (46), we investigated whether adjunctive treatment with GML or AM is able to augment the response to HAL. The inclusion of AM is 2-fold; to investigate whether any observed pharmacological effects of GML may be specific for one of the known bio-active constituents of the extract, i.e., AM, or whether these actions underscore a sum effect of the total extract and, second, to link any effects to a known psychotropic property of AM and/or GML. In order to cover the mood-psychosis continuum, behavioral analyses focused on positive (sensorimotor gating; locomotor hyperactivity) and negative (depression) related symptoms. Moreover, by measuring associated changes in plasma and brain redox-inflammatory markers, it explores possible activity within a key neuropathological feature of the illness, viz. immune-inflammatory dysfunction (35). This study has importance to the field in that a plant extract and one of its known bioactive constituents are compared to a reference control pharmaceutical agent across a range of behavioral and biological parameters of relevance to schizophrenia.

## Methods and Materials

### Chromatographic Fingerprinting of Raw GML

In order to determine the authenticity and constituents of GML, separation of prenylated xanthones found in GML was achieved utilizing reversed-phase high-performance liquid chromatography (HPLC) with diode-array detection (DAD) [see Oberholzer et al., (55)].

### Animals

Pregnant female Sprague–Dawley (SD) dams were used during the prenatal phase of the study. Male pups were weaned (PND 21) and used for the remainder of the study. Since this and our earlier paper (55) represent the first bio-behavioral studies evaluating the possible psychotropic benefits of GML in translational rodent models of neuropsychiatric illness, and that the hormone cycle of female rats is well known to influence the outcome of behavioral and pharmacological studies, e.g., Regenass et al. (59) and Harvey et al., (60), only male rats were used in the study.

In order to remove experimental bias, animals were randomly allocated by an experienced animal technologist blind to the study (61) to 12 rats per group (62). The number of rats per group was as directed by a statistical power analysis. Animals were bred, supplied, and housed at the Vivarium (South African Veterinary Council reg. no. FR15/13458; South African National Accreditation System good laboratory practice compliance no. G0019) of the Pre-Clinical Drug Development Platform of the North-West University (NWU) in identical cages containing corncob, under conditions of constant temperature (22 ± 1°C) and humidity (50 ± 10%) with a 12:12-h light/dark cycle (lights on 06:00 to 18:00). Food and water were provided *ad libitum* in the home cage, with corncob changed at least once a week. All experiments were approved by the AnimCare animal research ethics committee (National Health Research Ethics Council reg. no. AREC-130913-015) of the NWU. Animals were maintained, and all procedures performed in accordance with the code of ethics in research, training, and testing of drugs in South Africa and complied with national legislation (Ethical approval numbers: NWU-00376-16-A5 and NWU-00147-14-A5). The study design and procedures were according to the Animal Research: Reporting *in vivo* Experiments (ARRIVE) Guidelines (61).

### Study Design

**Figure 1:**
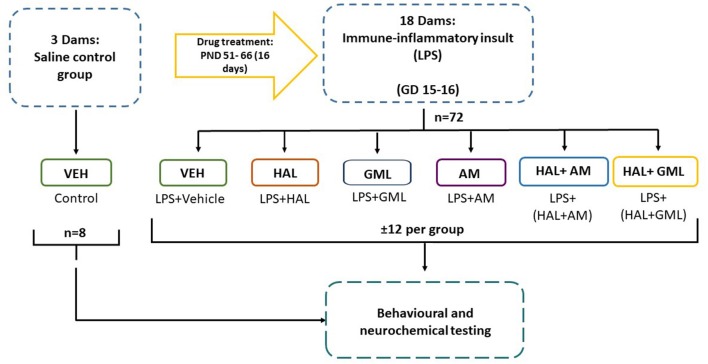
Schematic diagram of the study layout.

The exposure and treatment layout of the MIA model is presented in Figure 1. Treated dams (*n* = 18) received LPS from gestational days 15–16 with control dams (*n* = 3) receiving saline from gestational days 15–16. These GDs were chosen on the grounds of a previous study showing decreased fetal demise at this stage, as well as the correlation of this period with second trimester human pregnancy, suspected to be a critical period for the development of schizophrenia (63). Male offspring (±4 per dam) was used in the remainder of the study. A previous study did not demonstrate protective effects of cross-fostering in a MIA model (64). Off-spring was therefore not cross-fostered with healthy dams.

A total number of 72 male offspring from LPS-exposed dams (*n* = 72) were randomly divided into six treatment groups, each comprising 12 rats/group (65). These groups received oral dosing of the following: vehicle (saline, 1 ml/kg), HAL (2 mg/kg po) (66–69); GML (50 mg/kg po) (55), HAL + GML (HAL + GML) (at the previously mentioned doses), AM (20 mg/kg po) (70) and haloperidol + α-mangostin (HAL + AM) (at the previously mentioned doses) (Figure 1). Male offspring from the control dams (*n* = 8) received oral dosing of vehicle. The respective drug treatments continued for 16 days from PND 51–66 (55). During the last 2 days of treatment, all groups were subject to behavioral testing as follows: (1) prepulse inhibition (PPI) of startle on day 13 of treatment (PND 63), (2) the open field test (OFT) on day 14 of treatment (PND 64), and the forced swim test (FST) on day 14 of treatment (PND 64). The animals were euthanized 36 h later by decapitation with trunk blood and brain tissue collected and stored at −80°C for later neurochemical analysis.

### Drugs and Treatment

LPS (100 μg/kg) from *Escherichia coli* (*E. coli*) (Sigma-Aldrich, Johannesburg, South Africa) was dissolved in saline and administered subcutaneously (SC) to pregnant dams on GD 15–16 (30, 63). HAL (2 mg/kg/day; Sigma-Aldrich, Johannesburg, South Africa) was dissolved in a minimum volume of glacial acetic acid, then further diluted with distilled water and the pH adjusted using 10 N NaOH to 6–6.25 and administered by oral gavage (66). The dose of HAL was selected for oral dosing specifically and in line with an earlier study (71). The ground dried pericarp of GML fruit (Industrial Analytical, Kyalami, South Africa) was mixed in a 0.1% xanthan gum solution to aid suspension and administered by oral gavage, at a dose of 50 mg/kg/day (55). AM (Sigma-Aldrich, Castle Hill, Australia) was dissolved in polyethylene glycol (PEG) 400 vehicle (PEG 400:water ratio = 6:4, v/v) (72) and administered orally (20 mg/kg/day) (70).

### Behavioral Analyses

In order to assess whether the applied drug treatments are equally effective with respect to mood vs. psychosis-related manifestations of schizophrenia, PPI of startle (psychosis like), locomotor activity, and despair in the FST (depressive like) behaviors were assessed on PND's 64–65. Indeed, an earlier study found GML to be an effective antidepressant vs. imipramine using a genetic rodent model of depression (55). The current study design would not only re-affirm the earlier noted observation but do so in another translational model, while it would also possibly extend GML's scope of application to psychotic disorders like schizophrenia. This approach has validity since MIA not only evokes psychosis-like behavior in rat offspring (31–33) but also depressive-like manifestations (21), thereby presenting its suitability for studying broad pharmacological responses of relevance to schizophrenia.

### Prepulse Inhibition (PPI)

PPI is used to determine deficits in sensorimotor gating, well described in schizophrenia (73) and representative of cognitive fragmentation (74). PPI was assessed in illuminated and ventilated sound-attenuated startle chambers (SR-LAB, San Diego Instruments, San Diego, USA), as described previously (75). Startle amplitudes were defined as the average of 100 × 1-ms stabilimeter readings collected at stimulus onset. The stabilimeter was calibrated before each session.

Briefly, the startle session began with a 5-min acclimatization period, during which a 68-dB background noise level was maintained throughout the session; the basal startle response was then measured with 10 trials of a single 40-ms 120-dB white noise as a startle stimulus; after this, 80 trials of randomly delivered pulses, including 20 trials of 120 dB PULSE-ALONE trials, 50 PREPULSE trials (with intensities of 72, 76, 80, or 84 dB) and 10 trials with no pulse was delivered. A final 10 trials of single 40-ms 120-dB PULSE-ALONE startle stimuli was then supplied. After the testing session, the percentage PPI (%PPI) for the four pre-pulse intensities was calculated as %PPI = [100–(startle response for PREPULSE + PULSE trial)/(startle response for PULSE ALONE trial) × 100].

### Open Field Test (OFT)

The OFT was used to exclude any confounding locomotor effects of treatment in the FST (76). Moreover, motor activity in the OFT may be indicative of underlying neurotransmitter alterations, especially subcortical dopaminergic hyperactivity that has relevance to schizophrenia (77). Rats were tested individually in an open field arena (1 × 1 m), with total distance moved (cm) scored for 5 min using EthoVision XT® software (Noldus Information Technology, Wageningen, Netherlands).

### Forced Swim Test (FST)

The FST was used to screen for antidepressant-like properties following prenatal LPS exposure and drug treatment (78, 79). Negative symptoms of schizophrenia are closely related to depressive behavior (80), while schizophrenia is often co-morbid with major depression (81). The FST was performed as described previously (82), except the final swim was over a period of 7 min with the first and last minute discarded during analysis (55). Immobility time was scored as floating behavior with the rat maintaining only the necessary movements to keep its head above the water vs. escape-directed swimming (horizontal movements throughout the cylinder) and struggling or climbing (upward-directed movements in cylinder) behavior (76). The latter are noted for representing serotonergic and noradrenergic-mediated escape-directed behaviors, respectively (82). These behavioral components were recorded and scored on video by investigators blind to treatment, expressed in units of time (s). Behavior was scored using manual continuous timer software (FST Scoreboard 2.0 software; Academic Support Services: Information Technology in Education, NWU), previously validated against the traditional 5-s time-sampling technique (83).

## Neurochemical and Redox-Immune-Inflammatory Analyses

### Brain Tissue and Plasma Preparation

Thirty-six hours after the final behavioral analysis, rats were euthanized by decapitation, after which trunk blood was collected into pre-chilled, 4-ml vacutainer tubes (SGVac) containing dipotassium ethylenediaminetetraacetic acid (K_2_EDTA) solution as anticoagulant. Frontal cortex and striatum were dissected out on an ice-cooled glass slab as described previously (62, 84). Liquid nitrogen was used to fix the above brain regions and stored at −80°C until the day of analysis. The tissue was pre-split into aliquots for use in the different assays to avoid freeze–thaw–freeze changes and possible deactivation of components. On the day of assay, the tissue was weighed and allowed to thaw on ice. A 10% tissue homogenate was then prepared in a phosphate-buffered saline (PBS) using a Teflon homogenizer (84).

### Lipid Peroxidation Analysis

Thiobarbituric acid reactive substance (TBARS) is a by-product of lipid peroxidation. The Parameter™ TBARS assay from R&D Systems (Minneapolis, USA; catalog number KGE013) was used to analyze lipid peroxidation in brain tissue (45), according to the manufacturer's instructions. Absorbance was read at 532 nm using a Bio-Tek FL600 Microplate Fluorescence Reader (Bio-Tek, Instruments, Inc., 381 Highland Park, Winooski, VT, USA).

### Pro-inflammatory Cytokine Measurement

Plasma TNF-α was measured using the Rat TNF-α ELISA MAX™ Deluxe Set (catalog number 438204) from Bio Legend (San Diego, USA). IL-6 was measured using the Rat IL-6 ELISA Kit (catalog number E-EL-R0015) from Elabscience® (Wuhan, China). Both were performed in accordance with the manufacturer's instructions. Absorbance was read at 450 nm using the above noted instrument.

### Statistical Analyses

One-way factorial analysis of variance (ANOVA) and Bonferroni *post hoc* tests were used for the statistical analyses of FST scores, brain lipid peroxidation levels, and plasma cytokine analyses. For analysis of %PPI data, two-way ANOVA with repeated measures was used with Bonferroni *post hoc* tests. However, in order to compare the MIA model with the control group, an unpaired Student's *t*-test was used to analyze each parameter. To ensure there is complete equality of the variances of the differences between all variations of related groups, assumption of sphericity was conducted with Mauchly's test. If the assumption of sphericity was not met, the Greenhouse–Geisser correction was used. Normal distribution of the variables was assessed with a Q–Q plot and histogram for all variables in each treatment group. All data were normally distributed and expressed as the mean ± standard error of the mean (SEM), with a value of *p* < 0.05 considered statistically significant. Where additional detail was deemed useful, for example, when statistical significance was narrowly missed, a Cohen's d calculation was performed to establish effect size and practical significance: medium effect (0.5 ≥ d < 0.8), large effect (0.8 ≥ d < 1.3), and very large effect (d ≥ 1.3) sizes. Only large-to-very large effect sizes are presented in the figures and text. All data were analyzed and graphics prepared using GraphPad Prism 7, San Diego California, USA.

## Results

**Figure 2:**
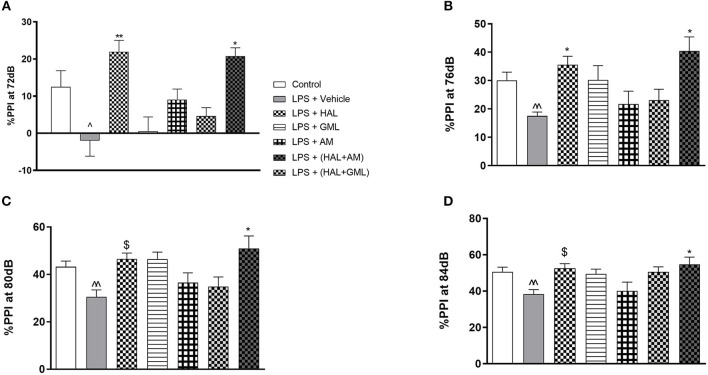
Sensorimotor gating with regard to percent prepulse inhibition (%PPI) of startle at **(A)** 72 dB, **(B)** 76 dB, **(C)** 80 dB, and **(D)** 84 dB in rats exposed to saline and treated with vehicle (Control) as well as rats exposed to LPS receiving vehicle and the various drug treatments as indicated [unpaired Student's *t*-test for Control vs. LPS + vehicle; ^∧^*p* < 0.05, ^∧∧^*p* < 0.01; two-way ANOVA with repeated measures for the different dB intensities, Bonferroni *post hoc* test; *p < 0.05, **p < 0.01 vs. LPS + Vehicle. ^*$*^*d* ≥ 1.3 vs. LPS + Vehicle (Cohen's *d* value)].

**Figure 3:**
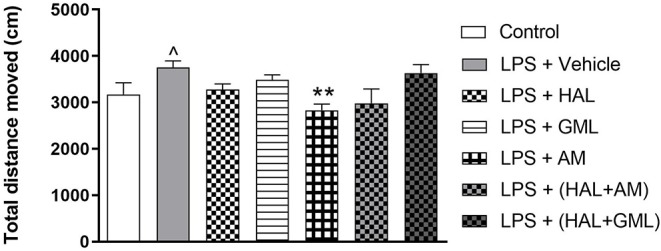
Locomotor activity (total distance moved in cm), analyzed in the OFT in rats exposed to saline and treated with vehicle (Control) as well as rats exposed to LPS receiving vehicle and the various drug treatments as indicated (unpaired Student's *t*-test for Control vs. LPS + vehicle; ^∧^*p* < 0.05; one-way ANOVA, Bonferroni *post hoc* test; **p < 0.01 vs. LPS + Vehicle).

**Figure 4:**
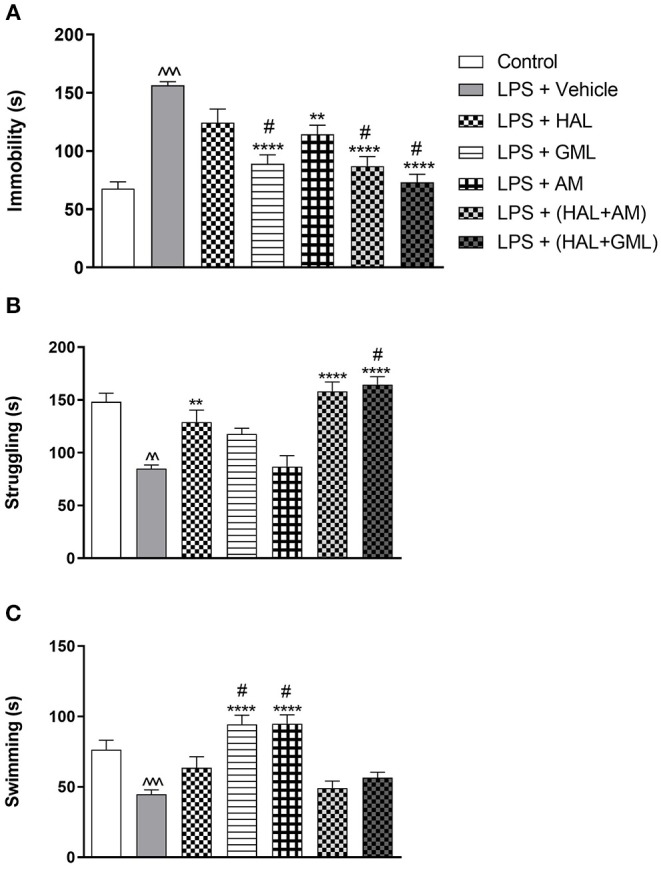
The forced swim test (FST) with regard to **(A)** immobility, **(B)** struggling, and **(C)** swimming behavior in rats exposed to saline and treated with vehicle (Control) as well as rats exposed to LPS receiving vehicle and the various drug treatments as indicated (unpaired Student's *t*-test for Control vs. LPS + vehicle; ^∧∧^*p* < 0.01, ^∧∧∧^*p* < 0.001; one-way ANOVA, Bonferroni *post hoc* test; **p < 0.01, ****p < 0.0001 vs. LPS + Vehicle; #*p* < 0.05 vs. LPS + HAL).

**Figure 5:**
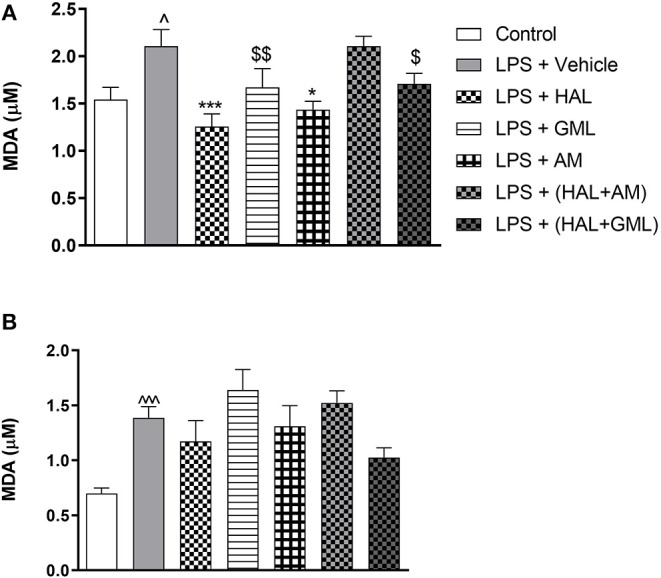
Lipid peroxidation as quantified by malondialdehyde (MDA) accumulation in **(A)** frontal cortex and **(B)** striatum in rats exposed to saline and treated with vehicle (Control) as well as rats exposed to LPS receiving vehicle and the various drug treatments as indicated [unpaired Student's t-test for Control vs. LPS + vehicle; ^∧^*p* < 0.05, ^∧∧∧^*p* < 0.0001; one-way ANOVA, Bonferroni *post hoc* test; *p < 0.05, ***p < 0.001 vs. LPS + Vehicle. ^*$*^*d* = 0.5 ≥ *d* < 0.8, ^*$$*^*d* = 0.8 ≥ *d* < 1.3 vs. LPS + Vehicle (Cohen's d value)].

**Figure 6:**
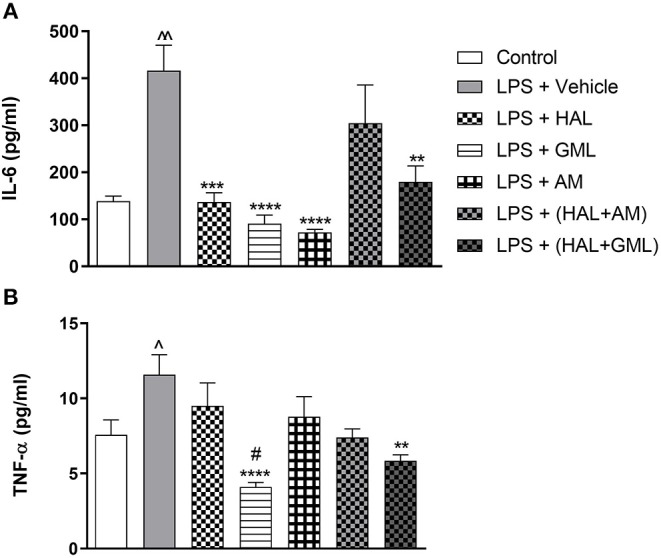
Plasma cytokine levels of **(A)** IL-6 and **(B)** TNF-α in rats exposed to saline and treated with vehicle (Control) as well as rats exposed to LPS receiving vehicle and the various drug treatments as indicated (unpaired Student's *t*-test for Control vs. LPS + vehicle; ^∧^*p* < 0.05, ^∧∧^*p* < 0.001; one-way ANOVA, Bonferroni *post hoc* test; **p < 0.01, ***p < 0.001, ****p < 0.0001 vs. LPS + Vehicle; #*p* < 0.05 vs. LPS + HAL).

MIA model validation, i.e., LPS vs. saline control, was analyzed separately using *T*-tests and presented in Figures 2–6. Thereafter, untreated LPS (MIA model) were compared to LPS plus the various drug treatments and analyzed separately using the appropriate ANOVA followed by *post hoc* Bonferroni analysis. The latter are also presented in Figures 2–6.

### GML Fingerprinting

A chromatogram of GML used in this study, and analyzed using reversed-phase HPLC with DAD, was found to contain predominantly α-mangostin (11.7%) and γ-mangostin (1.1%) (55).

## Treatment-Naive LPS- vs. Saline-Exposed Animals (MIA Model Validation) (Figures 2–6)

### Prepulse Inhibition of Acoustic Startle

When considering the LPS model alone compared to the vehicle control group, unpaired Student's *t*-tests revealed no significant differences between the groups at the respective startle blocks (data not shown).

Regarding %PPI and comparing the LPS-exposed group to the vehicle group using unpaired Student's *t*-tests, the LPS-exposed control group (LPS + vehicle) presented with significant deficits in %PPI at 72 dB (*p* = 0.0248), 76 dB (*p* = 0.003), 80 dB (*p* = 0.007), and 84 dB (*p* = 0.007) when compared to the control group (saline + vehicle) (Figures 2A–D).

### Open Field Test

Unpaired Student's *t*-test revealed a significant increase in locomotor activity in the LPS exposed group (LPS + vehicle) compared to the saline control group (saline + vehicle) (*p* = 0.039) (Figure 3).

### Forced Swim Test

Unpaired Student's *t*-test revealed a significant increase in immobility in the LPS-exposed rats (LPS + vehicle) when compared to the control group (saline + vehicle) (*p* < 0.0001) (Figure 4A). A significant decrease in both swimming (*p* = 0.0002) (Figure 4C) and struggling (*p* < 0.0001) (Figure 4B) behaviors was also observed in the LPS-exposed group (LPS + vehicle) compared to the control group (saline + vehicle).

### Regional Brain Lipid Peroxidation

Using unpaired Student's t-tests, frontal cortical malondialdehyde (MDA) levels were significantly increased in the LPS-exposed rats (LPS + vehicle) (*p* = 0.030), compared to the saline control group (saline + vehicle) (Figure 5A). In the striatum, significantly elevated levels of MDA were also observed in the LPS-exposed rats (LPS + vehicle) (*p* < 0.0001) in comparison with the saline control group (saline + vehicle) (Figure 5B).

### Cytokines

#### IL-6

Unpaired Student's *t*-test revealed that plasma IL-6 levels were significantly elevated in the LPS-exposed group (LPS + vehicle) when compared to the saline control group (saline + vehicle) (*p* = 0.0005) (Figure 6A).

#### TNF-α

Unpaired Student's *t*-test displayed significantly elevated plasma TNF-α levels in the LPS-exposed group (LPS + vehicle) when compared to their saline control group (saline + vehicle) (*p* = 0.041) (Figure 6B).

## LPS Model Plus Various Drug Treatments (Figures 2–6)

### Prepulse Inhibition of Acoustic Startle

Two-way ANOVA with repeated measures for each startle block in all the groups receiving the respective treatment or vehicle indicated a significant treatment × startle block interaction [*F*_(3, 73)_ = 67.69, *p* < 0.0001] but no significant main effect of startle block [*F*_(3, 73)_ = 1.62, *p* = 0.128] or treatment [*F*_(3, 73)_ = 1.023, *p* = 0.417] on startle amplitude (data not shown). Bonferroni *post hoc* testing on the startle amplitude in all the LPS-exposed groups receiving the respective treatments or vehicle indicated that all the groups had a significant decrease (*p* < 0.05) in startle amplitude from blocks 1 to 4 (data not shown). Bonferroni *post hoc* testing also revealed no significant differences between any of the exposed and treatment groups at the respective startle blocks.

When considering drug treatment in the LPS model (Figure 2), two-way ANOVA revealed a significant interaction between treatment and PPI intensity [*F*_(6, 77)_ = 295.66, *p* < 0.0001] as well as a significant main effect of treatment [*F*_(6, 77)_ = 5.63, *p* < 0.0001] and PPI intensity [*F*_(6, 77)_ = 1.83, *p* = 0.04] on %PPI in all the groups receiving the respective treatments or vehicle. Bonferroni *post hoc* testing demonstrated that HAL significantly reversed %PPI deficits in the LPS-exposed rats at 72 dB (*p* = 0.009) (Figure 2A) and 76 dB (*p* = 0.032) (Figure 2B). However, a very large effect size was observed in the LPS-exposed HAL-treated rats compared to their LPS vehicle-treated controls at 80 dB (*d* = 1.7) and 84 dB (*d* = 1.6) (Figures 2C,D, respectively). The combination treatment of GML + HAL successfully reversed %PPI deficits at all four of the prepulse intensities: 72 dB (*p* = 0.017), 76 dB (*p* = 0.002), 80 dB (*p* = 0.003), and 84 dB (*p* = 0.014) vs. the LPS-exposed control group (Figures 2A–D, respectively). However, no significant differences were observed in the LPS-exposed rats treated with GML alone, AM alone, or the combination of HAL + AM vs. the LPS + vehicle-exposed group (Figures 2A–D).

### Open Field Test

A one-way ANOVA of the OFT data in all the groups receiving the respective treatments or vehicle revealed a significant main effect of treatment on total distance moved [*F*_(6, 75)_ = 14.43, *p* < 0.0001]. When considering drug treatment in the LPS model, Bonferroni *post hoc* tests revealed no significant differences in the total distance moved in the LPS-exposed treatment groups receiving HAL, GML, HAL + GML, and HAL + AM when compared to the LPS-exposed control group (LPS + vehicle) (Figure 3). However, a significant decrease in locomotor activity was observed in the LPS-exposed group treated with AM (*p* = 0.006) compared to the control group (LPS + vehicle) (Figure 3).

### Forced Swim Test

A one-way ANOVA of all the groups revealed a significant main effect of treatment on immobility [*F*_(6, 77)_ = 16.02, *p* < 0.0001], struggling [*F*_(6, 77)_ = 15.44, *p* < 0.0001], and swimming [*F*_(6, 77)_ = 12.85, *p* < 0.0001]. When considering drug treatment in the LPS model (Figure 4), Bonferroni *post hoc* testing indicated a significant decrease in immobility in all the LPS-exposed treatment groups receiving GML (*p* < 0.0001), AM (*p* = 0.004), HAL + GML (*p* < 0.0001), and HAL + AM (*p* < 0.0001) compared to the LPS-exposed control group (LPS + vehicle) (Figure 4A). The effect of HAL alone did not reach significance (Figure 4A). However, treatment with GML alone (*p* = 0.034), HAL + GML (*p* < 0.0001), and HAL + AM (*p* = 0.03) showed a significantly greater decrease in immobility when compared to the HAL-treated LPS-exposed group (Figure 4A).

With regard to struggling behavior in the LPS-exposed rats, HAL (*p* = 0.005), HAL + GML (*p* < 0.0001), and HAL + AM (*p* < 0.0001) displayed a significant increase in struggling compared to the LPS-exposed control group (LPS + vehicle) (Figure 4B). The combination treatment of HAL + GML in the LPS rats displayed a significantly greater increase in struggling behavior (*p* = 0.049) when compared to HAL treatment alone in the LPS rats (Figure 4B).

Swimming behavior was significantly increased in the LPS groups receiving GML (*p* < 0.0001) and AM (*p* < 0.0001) treatment compared to the LPS-exposed control group (Figure 4C). A significant increase in swimming behavior was observed between the LPS-exposed group receiving HAL alone vs. both the LPS-exposed groups receiving GML (*p* = 0.005) or AM (*p* = 0.004), respectively (Figure 4C).

### Regional Brain Lipid Peroxidation

One-way ANOVA showed a significant main effect of treatment on lipid peroxidation in the frontal cortex [*F*_(6, 78)_ = 5.234, *p* < 0.0001] and the striatum [*F*_(6, 77)_ = 3.956, *p* = 0.002]. When considering drug treatment in the LPS model (Figure 5), Bonferroni *post hoc* analysis revealed that treatment with HAL (*p* = 0.001) and AM (*p* = 0.02) significantly reduced frontal cortical MDA levels in LPS-exposed rats, compared to the LPS-exposed control group (LPS + vehicle), but was unaffected by any of the other LPS-exposed treatment groups (LPS + GML, LPS + HAL + GML, and LPS + HAL + AM) vs. the LPS-exposed control group (Figure 5A). GML and GML + HAL showed a trend toward reducing MDA levels, with a large (*d* = 1.0) and medium (*d* = 0.7) effect size observed in the LPS-exposed rats treated with GML and GML + HAL, respectively, compared to their vehicle-treated controls (Figure 5A). Finally, none of the respective treatments showed a significant reduction in striatal MDA levels in the LPS-exposed rats compared to the LPS-exposed control group (Figure 5B).

### Cytokines

#### IL-6

One-way ANOVA revealed a significant main effect of treatment on IL-6 [*F*_(6, 76)_ = 5.93, *p* < 0.0001] in the LPS and vehicle-exposed and treated groups. When considering drug treatment in the LPS model (Figure 6A), Bonferroni *post hoc* analyses showed that treatment with HAL (*p* = 0.021), GML (*p* = 0.002), AM (*p* < 0.0001), and HAL + GML (*p* = 0.01) significantly reversed elevated levels of IL-6 in the LPS-exposed groups. However, HAL + AM had no significant effect on IL-6 plasma levels in the LPS-exposed animals compared to the vehicle control (Figure 6A).

#### TNF-α

One-way ANOVA revealed a significant main effect of treatment on TNF-α levels [*F*_(6, 77)_ = 5.96, *p* < 0.0001] in the LPS and vehicle-exposed groups receiving the respective treatments (Figure 6B). When considering drug treatment in the LPS model (Figure 6B), Bonferroni *post hoc* testing displayed that GML treatment successfully reversed elevated TNF-α levels in the LPS-exposed rats when compared to the LPS-exposed control group (LPS + vehicle) (*p* < 0.0001) (Figure 6B). In addition, GML was significantly more effective in decreasing plasma TNF-α levels in LPS-exposed animals than the HAL-treated LPS-exposed group (LPS + HAL) (*p* = 0.0081) (Figure 6B). Treatment with HAL + GML also significantly reduced TNF-α plasma levels in LPS-exposed animals in comparison to the LPS-exposed control group (*p* = 0.004) (Figure 6B). The remaining three treatment groups viz. HAL, AM, and HAL + AM showed no significant reduction in plasma levels of TNF-α when compared to the LPS-exposed control group (LPS + vehicle) (Figure 6B).

## Discussion

MIA induced sensorimotor gating deficits and depressive-like behavior concurrent with elevated cortico-striatal lipid peroxidation and elevated plasma pro-inflammatory cytokines in offspring. HAL reversed the changes in PPI, cortical (not striatal) lipid peroxidation, and elevated plasma IL-6, but failed to reverse depressive manifestations. GML + HAL effectively reversed PPI, while GML, AM, and HAL + AM did not. Conversely, GML and AM alone reversed depressive-like behaviors, more so than HAL, while the GML + HAL combination also reversed depressive-like symptoms. AM reversed both cortical (not striatal) lipid peroxidation and elevated plasma IL-6. GML and HAL + GML reversed elevations in IL-6 and TNF-α. These data present evidence for GML and its active constituent, AM, as being effective as adjunctive treatments but differently active with respect to psychotic and mood-related behaviors.

In line with previous findings (22, 31, 45, 63, 85), prenatal LPS exposure significantly compromised PPI in late adolescent offspring (Figure 2). HAL significantly reversed PPI deficits at 72 and 76 dB, with a similar trend and very large effect sizes at 80 and 84 dB (Figure 2), consistent with data from previous chronic (86) and acute (87–89) treatment studies in animals. Overactive dopaminergic processes are suggested to underlie the reduction in PPI (90), which explains the ability of HAL (D_2_ antagonist) to reverse said deficits (91). Although GML + HAL was effective in reversing MIA-induced PPI deficits across all four startle responses, this was not the case for GML or AM separately or for AM + HAL (Figure 2). The latter suggests that AM may abrogate the antipsychotic-like response to HAL, a particular interesting finding. In fact, AM presents with known antioxidant (58), 5HT2A, and a cAMP-PDE inhibitory (49) activity that to varying degrees may underlie these effects. Certainly, 5HT2A receptors play a prominent role in psychotic-like behavior (35) as well as in atypical antipsychotic drug design (35), and hence may play a role in the results described here. On the other hand, HAL is a potent pro-oxidant (92) and pro-inflammatory agent (93). Depending on the stage of disease progression, these actions, together with potent D2 inhibition, may mediate HAL's useful antipsychotic effects. However, these same pro-oxidative actions are purported to cause striatal toxicity and to cause late-onset treatment-related complications (92, 93). Although speculative, could these actions of HAL be countered by the antioxidant and cAMP-PDE inhibitory actions of AM, and possibly even reverse its antipsychotic effects? This opens up new ideas on how antipsychotics work and warrants further study.

Locomotor hyperactivity represents positive symptom schizophrenia, especially psychotic agitation (94). Prenatal LPS-exposed offspring demonstrated increased locomotor activity in the OFT (Figure 3), a behavioral response that has been ascribed to hyperdopaminergia (95). HAL lowered LPS-induced locomotor hyperactivity, albeit not significantly, in line with its antidopaminergic/antipsychotic actions. Only AM effectively reduced hyperlocomotion (Figure 3), yet it failed to alter PPI deficits (noted above). AM is a selective, competitive histamine antagonist (96) with sedative properties (97) that may adversely affect startle response in the PPI test. That said, the study design did not allow us to assess the effects of drug treatments on startle response in healthy controls, although rats exposed to LPS + AM did not differ significantly to rats exposed to LPS + vehicle. This and the above-noted HAL + AM findings prompt further research into the putative “antipsychotic-like” effects of AM. No other treatment had any noteworthy effects on locomotor activity (Figure 3), although the locomotor effects described for AM may complicate interpretation of swimming behavior in the FST (see below).

The negative symptoms of schizophrenia comprise affective flattening, alogia, anhedonia, asociality, and avolition (lack of motivation) (98), congruent with the basic symptoms of depression (99). Although the FST assesses behavioral despair, it has been suggested to signify an absence of motivational behavior which is commonly seen in schizophrenia (100). Indeed, LPS-exposed offspring presented with significant depressive-like behaviors (increased immobility) (Figure 4A) and reduced active coping (swimming and climbing) (Figures 4B,C), in agreement with earlier studies (101, 102). Especially, atypical antipsychotics may present with antidepressant activity (103, 104), while clinical (105) and preclinical (106) studies have described the pro-depressant effects of HAL (105, 106). In the present study, however, HAL showed a small, albeit negligible, effect to reverse MIA-associated immobility in the FST and reduced swimming (Figures 4A,C), although significantly increased struggling/climbing behavior (Figure 4B). Importantly, GML significantly decreased immobility and increased swimming behaviors (Figures 4A,C), antidepressant effects congruent with an earlier study in Flinders Sensitive Line (FSL) rats (55). Interestingly, the latter study described GML's prominent serotonergic actions and therapeutic equivalence with imipramine, which is shown here, too, but less emphatically (elevated swimming) (Figure 4C). Moreover, GML is a superior antidepressant to HAL (Figures 4A,C). Similarly, AM also exhibited significant antidepressant-like properties regarding its effects on immobility and swimming behavior, although not as marked as GML alone (immobility) (Figure 4A). Of note, GML and AM augmented the actions of HAL on immobility (Figure 4A) with GML bolstering the HAL effect on struggling (Figure 4B), suggesting a bolstering of HAL's action via mechanisms other than D_2_ receptor blockade. Despite AM suppressing locomotor activity, as noted above, this action did not affect its ability to reduce immobility and to increase swimming in the FST, thus highlighting a psychogenic action to bolster escape-driven behavior that is not related to, or mediated by, an increase in locomotor activity. Given the noted antioxidant actions of GML and its constituents, other antioxidants like NAC (107) are also antidepressant in the FST. Interestingly, NAC seems to have specific benefit in especially negative-symptom schizophrenia (40, 41), thus highlighting that antioxidants may have preferential psychopharmacological actions as antidepressants, which is borne out in this study as well.

Oxidative damage is implicated in the pathophysiology and neuroprogression of schizophrenia (35–37). Schizophrenia patients present with increased plasma lipid peroxidation (108–110), possibly correlated with certain clinical features (111). Adjunctive treatment with antioxidants improve symptoms in animal models (43, 112) as well as patients with schizophrenia (40). Prenatal LPS exposure significantly increased cortical and striatal lipid peroxidation in offspring (Figure 5), in agreement with previous findings (45, 113). Mouse models of oxidative stress are associated with cognitive and motivational deficits, as well as dysfunction of the prefrontal cortex (114). Schizophrenia is a hyperdopaminergic state (35) where dopamine metabolism contributes to oxidative stress by lowering glutathione (GSH) levels, which in turn is abrogated by D_1_/D_2_ receptor antagonists (36, 115). Importantly, HAL treatment significantly reduced lipid peroxidation in the frontal cortex (Figure 5A), while not having any marked effect in the striatum (Figure 5B). However, total striatum was analyzed here, whereas it is predominantly the ventral striatum encompassing the ventral tegmentum that is more relevant in rodents for an association with schizophrenia (116). The fact that HAL did not significantly reduce striatal lipid peroxidation may also be explained by HAL's known pro-oxidant actions in the striatum following chronic treatment (117) and which is associated with its long-term locomotor side effects. This action may otherwise obscure any possible antioxidant abilities in reducing MDA levels as was evident in the frontal cortex. This differential pro-oxidant action for HAL in these two brain regions is not new. In fact, Martins et al. (118) found that chronic HAL treatment increases oxidative stress in the striatum but decreases such levels in the cerebral cortex. Importantly, HAL *still* reversed LPS-induced PPI deficits (Figure 2), reiterating the importance of the frontal cortex in antipsychotic action (119). Moreover, here, we also show a frontal cortical role for its redox modulatory actions and how this may affect behavior. The frontal cortex is involved in cognitive processes such as working memory, behavioral flexibility, and attention (120). With regard to the antidepressant-like effects of GML and AM, the frontal cortex is also implicated in the development of depression and, hence, in antidepressant response (121, 122).

Although GML possesses antioxidant activity *in vitro* (123–126), GML had no effect on striatal lipid peroxidation, although it prompted a large effect size reduction in the frontal cortex (Figure 5A), thus qualitatively similar to that observed with HAL. Earlier, we found that chronic GML reversed elevated hippocampal lipid peroxidation in FSL rats (55), although the discrepancy between these two studies may be due to the different translational models used and the brain region assayed. AM also presents with antioxidant activity (58), here, significantly and again selectively reducing LPS-induced lipid peroxidation in the frontal cortex (Figure 5A). The absence of obvious antioxidant actions for GML and AM in the striatum is noteworthy, but may be related to assaying the whole striatum, as noted for HAL earlier. The diverse antioxidant actions of AM, viz. modulating GSH levels (127), free radical scavenging (128), inhibiting low-density lipoprotein oxidation (129), may afford it a more prominent antioxidant action than raw GML *in vivo*. As AM is the dominant bioactive xanthone in GML pericarp (55), it probably provides the dominant antioxidant activity observed with the raw extract. However, the more pronounced antioxidant action of AM does not translate into an improved behavioral outcome for AM over GML (Figures 2, 3), while GML and AM only offered small (GML) to negligible (AM) benefits in combination with HAL with regard to redox markers (Figure 5A). This suggests that GML may be offering beneficial effects through mechanisms other than antioxidant activity alone.

Immune-inflammatory dysfunction has been extensively reported in schizophrenia (35), specifically elevated levels of pro-inflammatory cytokines (130), while being a protagonist for oxidative stress (131). Pro-inflammatory cytokines have a developmental role in the brain (132, 133) and are implicated in the pathogenesis of neurodevelopmental disorders such as schizophrenia (134, 135). IL-6 and TNF-α levels are elevated in schizophrenia (136) and animal models (45, 137). Likewise, IL-6 and TNF-α were elevated in MIA offspring (Figures 6A,B) together with increased cortico-striatal lipid peroxidation (Figure 5). HAL-associated reversal of elevated IL-6 levels (Figure 6A) is consistent with clinical findings (138, 139), although it did not alter elevated plasma TNF-α levels (Figure 6B). Here, both GML and AM treatment reduced elevated plasma IL-6 levels (Figure 6A), with GML, but not AM, also reducing TNF-α levels (Figure 6B). This suggests a broader immunosuppressant action for GML vs. HAL or AM. AM has been shown to decrease inflammatory cytokines following LPS induction (140), to inhibit IL-2 release (141) and to suppress IL-6 expression (142). HAL + GML, but not HAL + AM, successfully reversed elevated IL-6 and TNF-α levels, although not more so than HAL alone (Figures 6A,B). In fact, HAL has immunosuppressive effects (143) as does GML have anti-inflammatory properties (125, 144). However, that neither GML nor AM bolstered the antioxidant effects of HAL again asserts that any beneficial effects offered by adjunctive GML treatment may involve mechanisms over and above inflammatory-redox processes. This warrants further study.

These findings have significance as a catalyst for future pre-clinical and clinical studies. Considering the important role of inflammation in the progression of mood and psychotic disorders, there is a growing interest in nutraceuticals with anti-inflammatory/antioxidant activity in psychiatry. That GML and AM have evinced therapeutic efficacy in the MIA model, as well as possess anti-inflammatory and antioxidant properties, suggests potential as a novel adjunctive treatment for these disorders (145). However, there appears to be distinct differential effects with respect to the mood–psychosis continuum, with a bias in favor of a depressed mood component. This prompts further investigation into GML's clinical benefits as an antidepressant vs. an antipsychotic. These aspects need deeper consideration in further animal studies but also in controlled clinical trials (146).

Certain limitations to this study are worth noting. Given the less-than-adequate antipsychotic-like effects for GML and AM, it would have been informative to include another schizophrenia-like behavioral assessment in the protocol to confirm these findings, e.g., memory, social interaction. Moreover, a dose titration analysis for GML and AM may have revealed a dose-dependent association in their behavioral effects, especially since the dose used for GML was based on a prior antidepressant study in another animal model (55). We also did not explore synergistic effects with atypical agents, which could differ to HAL. Biological analysis could have benefitted from regional striatal analysis, i.e., ventral, rostral, as opposed to assay of the whole striatum, as was done here. The study design did not allow for the assessment of drug treatments in healthy controls, which may have allowed for more in-depth explanation of treatment effects in LPS-exposed animals. Finally, having the same number of animals in both the saline-treated and LPS-exposed groups could have been an added benefit.

## Conclusion

Schizophrenia is plagued by poor treatment outcomes and the limited efficacy of currently available antipsychotics (147–149). Supplementary treatment with nutraceutical anti-inflammatory agents and antioxidants such as GML may offer distinct therapeutic benefits (46). Plant extracts invariably contain a rich mixture of various bioactive constituents, yet little is known whether the pharmacological properties of a given extract are the sum of one or a group of inherent constituents or the result of the unique mix that the raw extract offers. This study has attempted to highlight this important question. Unlike the reference antipsychotic, HAL, chronic treatment with GML or AM *failed* to impact on sensorimotor gating deficits in the MIA model. However, both GML and AM not only displayed significant antidepressant-like properties but also bolstered the anti-immobility response to HAL. This is noteworthy as unlike atypical antipsychotics, HAL is not a recognized antidepressant, while here, it only marginally reduced immobility in the FST. GML and AM were both anti-inflammatory in the model, which may underlie their antidepressant effects. AM and, to a lesser degree, GML abrogated frontal cortical oxidative stress. This study confirms the antidepressant-like effects of GML described in another translational model of depression, the FSL rat (55). Having performed this study in a MIA model supports the use of GML and AM to address depressive symptoms in schizophrenia. However, their ability to address broader psychotic manifestations of the illness, e.g., PPI deficits, requires further study. Whether GML or AM is able to confer therapeutic benefits remains to be confirmed in dose–response and other clinical studies (150).

## Data Availability Statement

The datasets generated for this study are available on request to the corresponding author.

## Ethics Statement

The animal study was reviewed and approved by AnimCare animal research ethics committee (National Health Research Ethics Council reg. no. AREC-130913-015) of the North-West University (Ethical approval numbers: NWU-00376-16-A5 and NWU-00147-14-A5).

## Author Contributions

JL: all laboratory work, data collection and formal analysis, validation, and first draft of the manuscript. MM: all figures, supervised behavioral methods and analysis, and statistical analysis. MB and OD: study design, data interpretation, and manuscript review. BH: conceptualization, methodology and statistics, writing—review and editing, supervision of JL, project administration, and funding acquisition.

### Conflict of Interest

Over the past 3 years, BH has participated in advisory boards, received honoraria from Servier, and received research funding from Servier, Lundbeck, Deakin University, Cannabis Science Inc., and HG&H Pharmaceuticals. The remaining authors declare that the research was conducted in the absence of any commercial or financial relationships that could be construed as a potential conflict of interest.

## Abbreviations

AM: α-mangostin
ANOVA: analysis of variance
ARRIVE: Animal Research: Reporting *in vivo* Experiments
cAMP: cyclic adenosine monophosphate
DAD: diode-array detection
FST: forced swim test
GML: *Garcinia mangostana* Linn
HAL: haloperidol
HPLC: high-pressure liquids chromatography
IL: interleukin
LPS: lipopolysaccharide
MDA: malondialdehyde
MIA: maternal immune activation
NAC: N-acetyl cysteine
NWU: North-West University
OFT: open field test
PBS: phosphate-buffered saline
PDE: phosphodiesterase
PEG: polyethylene glycol
PND: post-natal day
Po: *per os*
Poly I:C: polyinosinic, polycytidylic acid
PPI: prepulse inhibition
SD: Sprague–Dawley
TBARS: thiobarbituric acid reactive substance
TNF: tumor necrosis factor.
